# AI-Driven Nondestructive Measurement Technologies for Meat Quality and Safety: A Review

**DOI:** 10.3390/foods15152592

**Published:** 2026-07-24

**Authors:** Lorna Bridget Alal, Juntae Kim, Yun-Kil Kwon, Sun-Moon Kang, Isa Kabenge, Byoung-Kwan Cho

**Affiliations:** 1 Department of Smart Agriculture Systems, College of Agriculture and Life Sciences, Chungnam National University, Daejeon 34134, Republic of Korea; lorna.b.alal12@gmail.com; 2 Department of Smart Agriculture Systems Machinery Engineering, College of Agriculture and Life Sciences, Chungnam National University, Daejeon 34134, Republic of Korea; biosch94@gmail.com; 3 XCore System Co., Ltd., Sejong 30141, Republic of Korea; ykkwon@xcs.co.kr; 4 Animal Products Research and Development Division, National Institute of Animal Science, Wanju 55365, Republic of Korea; smkang77@korea.kr; 5 Department of Agricultural and Biosystems Engineering, College of Agricultural and Environmental Sciences, Makerere University, Kampala P.O. Box 7062, Uganda; isakabenge@gmail.com

**Keywords:** meat quality, meat safety, nondestructive techniques, artificial intelligence, industrial deployment

## Abstract

Meat quality and safety are critical aspects of global food security. However, traditional evaluation techniques, including sensory analysis and chemical and instrumental tests, are constrained by subjectivity, high time consumption, their destructive character, and susceptibility to bias. With rapid advances in sensor technologies and computational methods, there is a growing demand for the nondestructive, accurate, and fast measurement of meat quality and safety attributes. In recent years, artificial intelligence (AI) integrated with nondestructive sensing has emerged as a transformative paradigm, offering unparalleled capabilities for extracting quality information from complex datasets generated by various nondestructive sensing technologies. This review provides a comprehensive analysis of AI-driven nondestructive technologies for meat quality and safety assessment, focusing on the integration of machine learning and deep learning with various sensing techniques. Additionally, the review evaluates state-of-the-art algorithms and their performances and identifies deployment barriers, particularly calibration transfer, environmental sensitivity, reproducibility issues, sensor fouling, and generalization challenges across batches and processing plants. Furthermore, economic and regulatory constraints, including high sensor costs, small and medium enterprise (SME) adoption challenges, and alignment with HACCP/ISO frameworks that further limit commercial scalability, are discussed. Unlike previous reviews that primarily focus on individual sensing techniques, this review emphasizes the practical challenges associated with industrial implementation and the development of scalable solutions for real-world deployment. Finally, strategic research priorities and recommendations are highlighted to accelerate the industrial adoption of intelligent meat quality monitoring systems across the global meat industry.

## 1. Introduction

Global meat consumption has increased significantly in recent decades, driven by population growth, urbanization, and rising disposable incomes [1]. According to the Organization for Economic Co-operation and Development (OECD) and the Food and Agriculture Organization of the United Nations (FAO), global per capita meat consumption increased from 42.89 kg/year in 2019 to 45.12 kg/year in 2022 [2]. Meat remains a major source of protein, lipids, vitamins, and essential minerals, but it is prone to contamination by pathogens or chemical residues, which threaten public health and undermine consumer confidence. Therefore, reliable and efficient quality and safety assessments are critical [3]. Traditional evaluation approaches, including subjective sensory assessments and objective laboratory-based analyses, while rapid and inexpensive in some instances, are inadequate for modern processing demands. Sensory tests are prone to bias and lack standardization [4], while laboratory techniques are destructive, time-consuming, reagent-intensive, and unsuitable for real-time monitoring in high-throughput processing lines [5,6].

To overcome these limitations, rapid, nondestructive sensing technologies have emerged as promising alternatives for assessing meat quality and safety attributes. Techniques such as computer vision systems, electronic nose systems, and spectroscopic and imaging methods enable rapid, reagent-free assessment of physical and chemical attributes without damaging the sample [4,7]. When properly deployed, they enable continuous monitoring on processing lines. However, these sensors produce large, complex datasets, creating a need to convert these raw signals into actionable quality or safety metrics. The integration of artificial intelligence plays a transformative role by analyzing high-dimensional sensor outputs and extracting meaningful patterns for decision-making. Consequently, AI facilitates the translation of raw data into operational decisions. AI models can identify complex nonlinear relationships within imaging and spectroscopic data, enabling the accurate prediction of compositional attributes, freshness, and contamination. Convolutional neural networks have been applied to marbling and freshness assessments [8,9], while near-infrared spectroscopy combined with partial least squares regression has proven effective for predicting the moisture, fat, and protein content [10,11,12]. Electronic nose systems integrated with machine learning further enable the detection of volatile organic compounds associated with spoilage [13,14].

**Figure 1:**
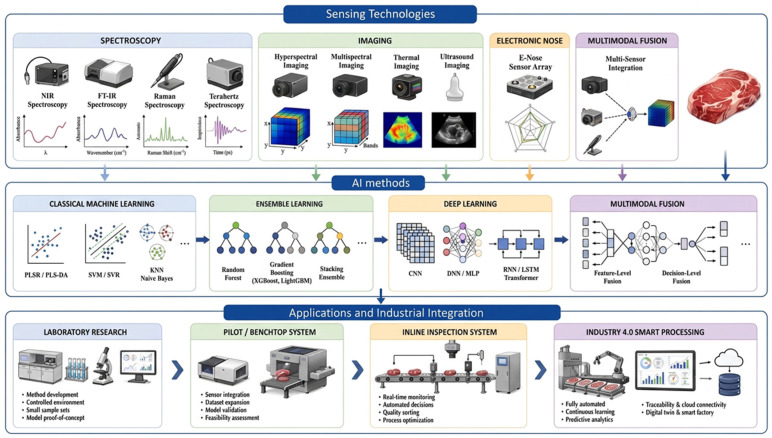
Conceptualization of nondestructive techniques, AI-based data analysis approaches, and industrial deployment progressive stages.

Beyond improving detection accuracy, AI-driven systems offer predictive capabilities such as spoilage forecasting, process optimization, shelf-life estimation, and waste reduction [15,16,17]. Despite these advances, most AI-enabled sensing solutions are still confined to the laboratory or pilot stage. Integration into industrial meat processing faces additional challenges, including high integration costs, stringent food-safety regulations, and the variability of raw materials and packaging. Existing studies frequently emphasize model development and prediction performance, while comparatively less attention has been given to real-world deployment factors. This review addresses this gap by linking sensors and AI algorithms directly to industrial deployment limitations and challenges. In this review, recent advances in both sensing modalities and artificial intelligence models with a focus on industrial applicability have been synthesized. This includes evaluating practical challenges, such as equipment readiness, regulatory acceptance, and data management, that are often overlooked in academic studies. By highlighting both technological developments and real-world constraints, we aim to guide researchers and industry stakeholders alike in advancing truly deployable, AI-driven nondestructive assessment systems. Figure 1 presents a conceptual overview of nondestructive meat quality and safety assessment techniques, AI-based data analysis approaches and progressive stages that build up to industrial deployment.

The remainder of this manuscript is organized as follows: Section 2 provides the literature search strategy while Section 3 provides an overview of the major nondestructive sensing technologies used for meat quality and safety evaluation. AI approaches, including machine learning and deep learning methods for analyzing data generated by these sensing systems are discussed in Section 4. The review delves into the challenges and limitations associated with the industrial implementation of AI-driven sensing technologies in Section 5 and finally outlines future research directions and strategies for advancing scalable AI-enabled meat quality monitoring systems in Section 6.

## 2. Literature Search Strategy

**Figure 2:**
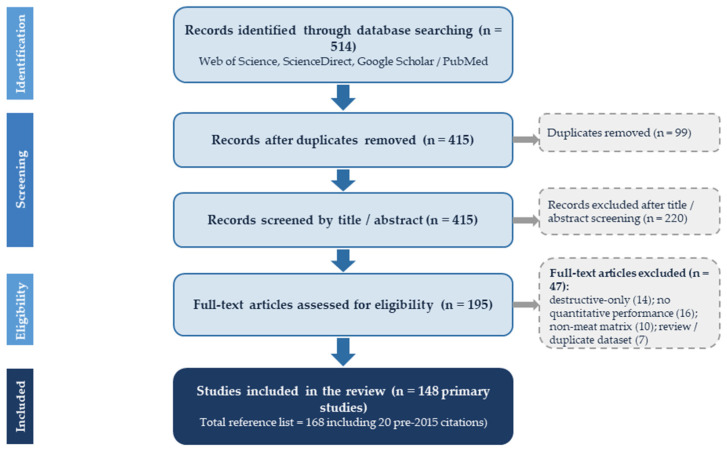
PRISMA flow diagram summarizing the identification, screening, and inclusion of studies in this review.

The literature underpinning this review was retrieved from Web of Science, ScienceDirect, and Google Scholar, supplemented by PubMed for microbiological and food-safety records. The systematic search covered January 2015–April 2026, with search strings combining sensing-technology terms (near-infrared, hyperspectral, Raman, terahertz, fluorescence, computer vision, electronic nose, multispectral, ultrasound, and X-ray/CT/DEXA), meat-matrix terms (meat, beef, pork, poultry, and lamb), quality and safety attributes (freshness, TVB-N, microbial load, adulteration, marbling, and composition), and analytics terms (machine learning, deep learning, chemometrics, and calibration transfer), joined using Boolean AND/OR operators. This search identified 514 records, which were screened following a Preferred Reporting Items for Systematic Reviews and Meta-Analyses (PRISMA) 2020 guidelines, after duplicate removal and sequential title, abstract, and full-text screening against the inclusion criteria; 148 studies from the defined search window were retained (Figure 2). The completed PRISMA 2020 checklist is provided in the Supplementary Materials [18]. In addition, a limited number of foundational and landmark methodological references dating back to 2004 (for example, early support vector machine, near-infrared, and partial least squares studies) were incorporated through targeted backward and forward citation tracking, where necessary to explain the conceptual or algorithmic origin of a given sensing technique. Combining these 20 foundational references with the 148 studies identified through the systematic search yielded a total of 168 sources synthesized in this review.

## 3. Nondestructive Technologies

### 3.1. Computer Vision Systems (CVSs)

Computer Vision Systems (CVSs) have emerged as powerful tools, enabling the rapid, nondestructive evaluation of meat quality and safety attributes, including color, shape, size, and surface texture [1]. A typical CVS setup comprises an illumination source, a charge-coupled device (CCD) camera, a frame grabber, and a processing unit [2]. These systems capture digital images under controlled illumination, extract quantitative features through image processing algorithms, and correlate these features with quality parameters using AI models. CVS can quantify attributes that appear visibly. Surface color, marbling, and color-based defects, such as Pale Soft Exudative (PSE) and Dark Firm Dry (DFD), tend to be captured well. Current systems employ multiple color spaces, with CIELAB, RGB, and HSV/HSI being most prevalent. Chmiel et al. [3] reported a correlation coefficient of R = −0.80 between the L* component and pH for DFD beef detection, with lightness components from HSV and HSL achieving 90.0% and 88.4% classification accuracy, respectively. The negative R values reflected lower lightness, associated with higher-pH DFD beef. Similarly, Taheri-Garavand et al. [2] achieved R = 0.98734 (MSE = 0.002045) for chicken freshness assessment through multivariate analysis across RGB, HSI, and CIELAB color spaces.

CVS performance varies systematically by quality attribute, reflecting the strength of surface-to-internal correlations. For freshness classification, color oxidation provides a reliable visible representation, with Barbri et al. [4] reporting 95.24% accuracy and B. Wang et al. [5] achieving R^2^p = 0.970 and R^2^p = 0.993 for TVB-N and TBA, respectively, in chicken thighs. Marbling assessment also reaches deployable accuracy. Barbon et al. [6] achieved 76.1% and 81.6% with k-nearest neighbors (k-NN) for pork and beef, while X. Sun et al. [7] graded pork loin quality using SVM, predicting marbling and color scores at 69.6% and 78.9% accuracy, respectively. However, CVS struggles with attributes lacking unique visible signatures, such as protein and moisture content, where the authors of [8] reported weak correlations of R^2^p = 0.26 and 0.43, with RMSEP values of 1.93 and 2.00, respectively.

Overall, CVS offers high speed, low cost, and suitability for inline grading, as cameras can operate at conveyor speeds with modest hardware requirements. However, performance depends heavily on controlled illumination and clean surfaces. Glare, uneven lighting, variable datasets, and viewing angles can distort extracted features. Thus, CVS is well-suited for appearance-based grading but cannot reliably assess safety or internal composition [9,10]. Detection of pathogens, toxins, or internal fat distribution necessitates spectral techniques.

### 3.2. Spectroscopic Techniques

#### 3.2.1. Near-Infrared (NIR) Spectroscopy

Near-infrared (NIR) spectroscopy addresses the surface limitations of computer vision by probing molecular constituents within meat. Operating in the 780–2500 nm range (12,800–4000 cm^−1^), NIR detects overtone and combination bands from C–H (lipids), N–H (proteins), and O–H (water) vibrations [11,12]. Absorption of NIR radiation excites higher vibrational states, generating wavelength-specific spectral features whose intensity and position enable quantitative estimation of moisture, protein, and fat [13]. This subsurface penetration provides chemical information inaccessible to surface imaging.

The defined absorption mechanisms support strong quantitative predictions of major constituents, with RPD values above 2.01 and R^2^cv values above 0.90 reported for beef and pork [14,15,16]. In contrast, pH and color rely on indirect associations with water holding capacity rather than specific chromophores, resulting in moderate performance (RPD < 2.3 and R^2^cv = 0.67–0.86) that often declines under external validation [14,17,19,20]. NIR also supports adulteration detection. Barbin et al. [21] achieved 75–100% classification accuracy for turkey authenticity using LDA. However, microbial quantification remains less robust. Arias et al. [22] reported cross-validated TVC prediction of R^2^cv = 0.897 (RMSECV = 0.992), which decreased to R^2^p = 0.694 (RMSEP = 1.037) under external validation, reflecting dependence on metabolic representations rather than direct bacterial signals. Additional NIR applications are summarized in Table 1.

Despite its strengths, NIR faces practical constraints. Penetration depth in dense tissue is limited, and weak absorbers reduce sensitivity, increasing detection limits [23,24]. Spectra are influenced by temperature-induced band shifts, surface moisture saturation, and fiber-optic transmission losses that lower signal-to-noise ratios in inline systems [25,26]. Moreover, instruments remain relatively costly, and calibration transfer across devices or facilities requires rigorous standardization [27].

#### 3.2.2. Fourier Transform Infrared (FT-IR) Spectroscopy

Fourier Transform Infrared (FT-IR) spectroscopy in the mid-infrared region (4000–400 cm^−1^) is widely applied to evaluate meat freshness, spoilage, adulteration, and compositional quality [28]. Unlike dispersive IR, FT-IR employs an interferometer that captures all frequencies simultaneously as a time-domain interferogram, which is converted into an absorbance spectrum using Fourier transform [29]. The resulting spectrum provides a highly specific molecular fingerprint. The integration of Attenuated Total Reflectance (ATR) enhances surface sensitivity and reproducibility, improving suitability for heterogeneous meat matrices [30].

Recent studies confirm strong performance in authenticity and microbial assessment. The authors of [30] combined ATR-FT-IR with PLSR and SVM to authenticate beef and pork, achieving 97% accuracy and detecting adulteration down to 5%, with lipid (1740 cm^−1^) and protein (1650 cm^−1^) bands serving as key discriminators. For spoilage evaluation, Dourou et al. [31] developed SVM-based models predicting microbial load in chicken liver (R^2^p = 0.708–0.828, RMSEP = 0.664–0.949 log CFU g^−1^). Similarly, Fengou et al. [32] reported 79.4–89.7% external validation accuracy for microbiological quality classification. Additional applications are summarized in Table 1.

Despite these advantages, limited MIR penetration depth (approximately 1–5 µm) restricts FT-IR largely to surface characterization, reducing reliability for bulk attributes in samples with heterogeneous moisture or fat distribution [28,29]. Spectral overlap between water and protein bands further complicates quantitative analysis. Moreover, sensitivity to temperature and humidity requires controlled measurement conditions, limiting field-scale implementation.

#### 3.2.3. Raman Spectroscopy

**Figure 3:**
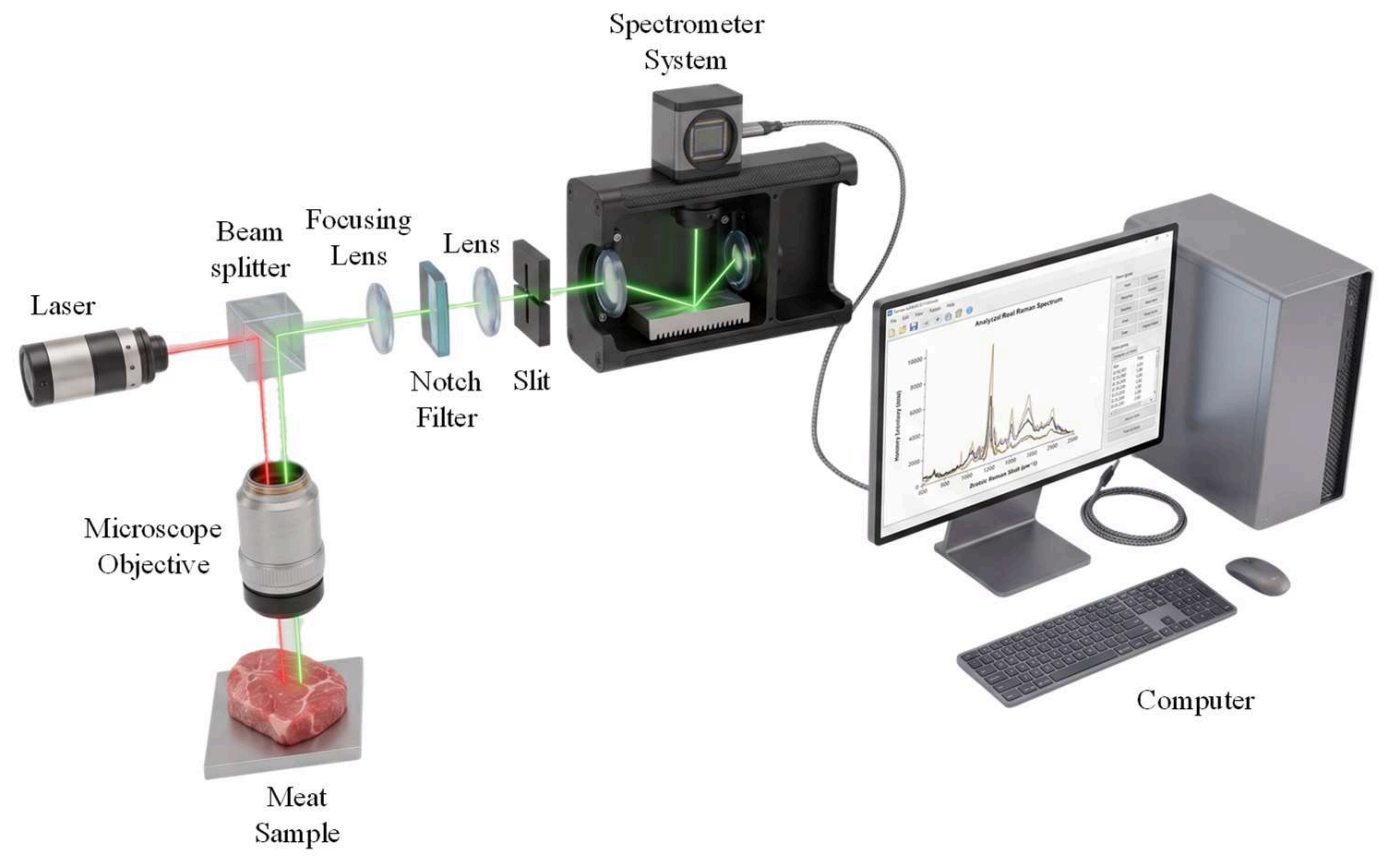
Schematic diagram of a typical Raman spectrometer.

Raman spectroscopy evaluates meat quality by detecting molecular vibrations through monochromatic laser interactions, generating sample-specific spectral fingerprints [33]. A small fraction (1 in 10^8^) of incident photons undergo inelastic scattering, generating Stokes and anti-Stokes Raman shifts that correspond to molecular vibrational modes [34,35,36]. Unlike infrared spectroscopy, Raman depends on changes in molecular polarizability, making nonpolar bonds (C=C, C–C, S–S) particularly responsive [37]. Its low sensitivity to water interference enhances suitability for high-moisture meat systems with minimal sample preparation. Figure 3 illustrates the components of a typical Raman spectroscopic system.

Raman spectroscopy has demonstrated value for selected meat quality traits. For example, Scheier et al. [38] used a handheld probe at the slaughter line to predict early post-mortem pH and drip loss, achieving R^2^cv = 0.75 (RMSECV = 0.09) for pH_35_ and R^2^cv = 0.83 (RMSECV = 0.6%) for drip loss. Lipid-related attributes are particularly well predicted. Shoko et al. [39] reported PLSR models for fatty acids with R^2^cv up to 0.85–0.90 (palmitic acid: R^2^cv = 0.80; oleic acid: R^2^cv = 0.71; docosadienoic acid: R^2^cv = 0.85; RMSECV = 0.7–4.5%). Similarly, X. Liu et al. [40] achieved 72.1% accuracy for detecting boar taint in porcine backfat using PLS-DA, linking spectral features to fat unsaturation and crystallinity differences. In contrast, the prediction of protein content and texture is less robust. Zhao et al. [41] reported good performance for flavor prediction in aged longissimus thoracis (R^2^cv = 0.80–0.84; RMSECV = 4.21–4.65) but weaker results for texture parameters (R^2^cv = 0.45–0.55), reflecting the complex biochemical basis of mechanical properties.

Overall, Raman’s strength lies in identifying specific molecular components, particularly lipids and related compounds [36,42]. However, its point-based measurement captures only localized information, making it poorly representative of heterogeneous meat structures and leading to high variability between scans. This undermines model stability, reproducibility, and accuracy. Additionally, slow acquisition times reduce throughput, making full-sample scanning impractical in industrial settings. Fluorescence interference from intrinsic compounds further degrades signal quality and complicates calibration, while high equipment costs and operational complexity limit scalability in processing environments.

The authors of this review view Raman as a specialized rather than a general purpose solution. It is most valuable for targeted compositional analysis, where its molecular specificity is advantageous. However, it is less suitable for high-speed, inline grading tasks. Practical deployment is therefore better suited to controlled environments like laboratories or end-of-line quality checks. While advancements such as fluorescence suppression and imaging approaches may improve performance, challenges in throughput and robustness remain significant barriers to widespread industrial adoption.

#### 3.2.4. Terahertz Spectroscopy

**Figure 4:**
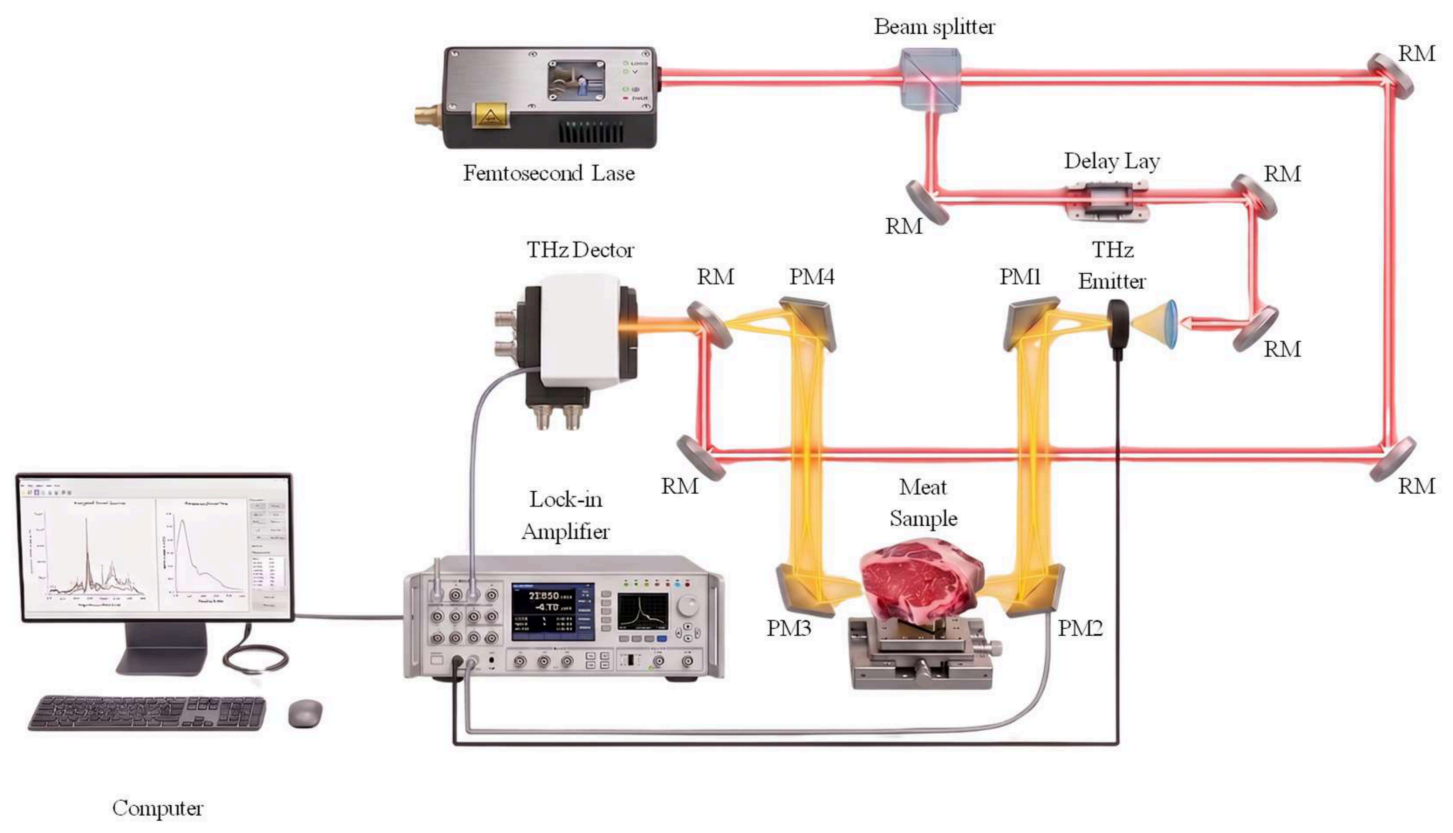
Schematic diagram of a typical Terahertz spectroscopic system; Abbreviations: PM: parabolic mirror, RM: reflective mirror.

Terahertz (THz) spectroscopy is an emerging tool in meat quality and safety assessment, though current applications remain limited. Operating in the 0.1–10 THz range, THz waves probe polar molecular motions and collective dynamics without inducing bond breakage or exciting high-energy vibrations [43,44]. Their low photon energy (0.4–40 meV) enables sensitivity to rotational and lattice motions, particularly in polar structures [45]. Two main modalities are used: time-domain (THz-TDS), which captures broadband temporal responses suitable for monitoring compositional changes, and frequency-domain (THz-FDS), which offers high spectral resolution for targeted contaminant detection [45]. A typical Terahertz system is illustrated in Figure 4.

A key advantage of THz spectroscopy is its non-ionizing penetration through packaging materials such as paper and plastics, enabling inspection of sealed products [46]. Its sensitivity to structural and dielectric contrasts supports the detection of foreign bodies. C. Wang et al. [47] demonstrated 98.3–100% accuracy in identifying metal contaminants (1.4–6 mm) in sausages using discriminant analysis. THz has also shown potential for freshness evaluation. Liang et al. [44] applied THz-TDS to predict pork K-value, achieving RMSEP = 0.098 and R^2^p = 0.84 with an AdaBoost-optimized BP-ANN model. Spectral features at 0.8–1.2 THz correlated with ATP degradation products, indicating sensitivity to nucleotide changes during spoilage. Additional applications are summarized in Table 1.

While terahertz spectroscopy shows promising laboratory performance, it faces major limitations that hinder real-world deployment. Its most critical constraint is strong water absorption, which severely limits penetration depth in high-moisture materials like meat, restricting analysis to only thin surface layers [43]. Additionally, THz systems are expensive, sensitive to environmental conditions, and prone to signal instability and scattering in complex samples [44,48]. Limited spectral databases and a lack of standardization collectively pose significant barriers to industrial scalability, further reducing reliability.

In summary, THz remains an immature technique, best suited for research or niche applications rather than routine industrial use. It may be useful in controlled environments, such as detecting contamination in packaged or low-moisture products, but it is not yet viable for inline meat inspection or grading.

#### 3.2.5. Fluorescence Spectroscopy

**Figure 5:**
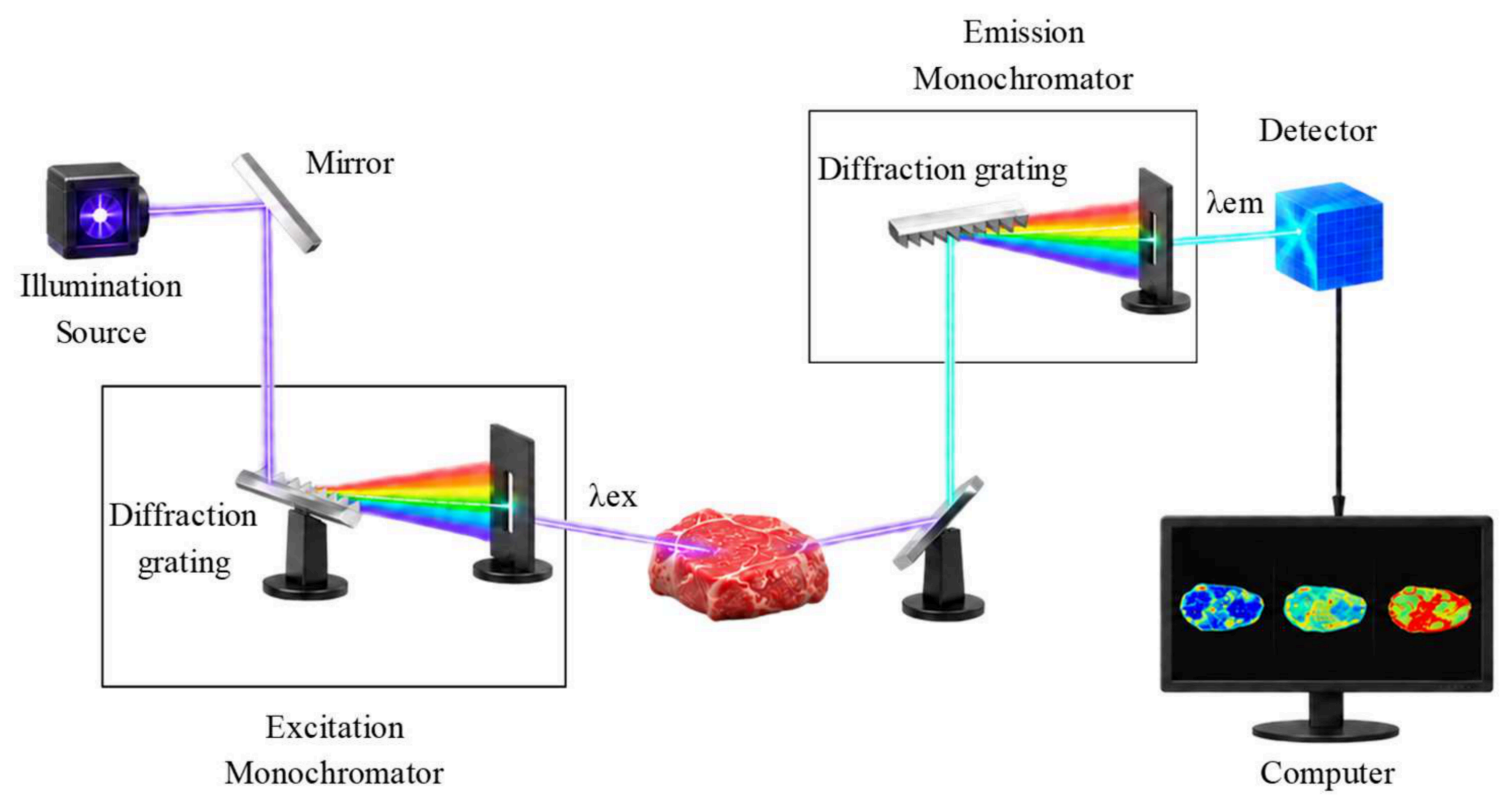
Schematic diagram of a typical fluorescence spectrometer.

Fluorescence spectroscopy detects intrinsic fluorophores by exciting samples with UV or visible light and measuring longer wavelength emissions generated during relaxation to the ground state [49]. These emissions form spectral fingerprints linked to quality and safety parameters, typically expressed as excitation or emission spectra. An illustration of the main components of a fluorescence system is demonstrated in Figure 5. Two main approaches are used: Synchronous Fluorescence Spectroscopy (SFS), which scans excitation and emission simultaneously to sharpen spectral features [49], and Excitation-Emission Matrices (EEM), which record emission spectra across multiple excitation wavelengths to generate fluorescence contour maps [50,51].

Fluorescence is particularly effective for freshness and spoilage markers associated with specific fluorophores. The authors of [52] applied EEM with a parallel factor analysis (PARAFAC) to monitor beef freshness, correlating NADH (320/445 nm) and Schiff base (380/470 nm) peaks with TVB-N and TBARS, achieving 93.33% validation accuracy. Similarly, Liu et al. [53] reported strong PLS performance for TVB-N and TBARS (R^2^p above 0.900 and RMSEP below 0.498) and for TVC (R^2^p = 0.871 with RMSEP = 0.399). Schlosser et al. [54] further demonstrated handheld probe applicability for minced pork freshness, achieving R^2^cv = 0.90–0.95 and R^2^p = 0.83–0.88. Beyond freshness, Aït-Kaddour et al. [55] used FFFS and SFS with machine learning (PLS-DA, PLS-SVM, PCA-SVM) to discriminate beef muscles, with FFFS-PLS-SVM reaching 94.34% recall, reflecting sensitivity to metabolic muscle differences. More studies are summarized in Table 1, while Table 2 provides a quick overview of evaluation metrics commonly used with machine learning and deep learning models for better interpretation of model performances.

Fluorescence spectroscopy offers fast, sensitive measurements but is fundamentally limited by its dependence on intrinsic fluorophores. It can only detect compounds that emit fluorescence, leaving key bulk attributes like fat, water, and total protein unmeasured. Aït-Kaddour et al. [56] reported only moderate predictive performance for fatty acids using FFFS (saturated: R^2^p = 0.66 and RPD = 2.29; monounsaturated: R^2^p = 0.48 and RPD = 1.49), and polyunsaturated fatty acids remained difficult to model due to low abundance and structural complexity. Furthermore, signal overlap and masking among dominant fluorophores further complicate analysis [57,58]. Additionally, its shallow penetration depth restricts measurements to surface layers, making it unsuitable for heterogeneous meat structures. These constraints affect deployment by limiting accuracy, robustness, and applicability across varying products and processing conditions.

Consequently, fluorescence is a practical but specialized tool. Its speed, low cost, and strong performance in detecting spoilage indicators make it suitable for targeted, real-time quality control applications. However, it is not a comprehensive solution for meat analysis and cannot replace broader compositional techniques like NIR or Raman. Effective deployment requires careful calibration, selection of excitation wavelengths, and integration into multi-sensor systems. Overall, fluorescence is best applied to specific tasks where clear fluorescent markers exist, rather than as a universal inline sensing technology.

**Table 1:** Summary of spectroscopic applications in meat quality and safety.

Technique	Meat Type	Quality Attributes	Acquisition Mode	Analytical Approach	Model Performance	Reference
NIR	Beef (longissimus thoracis muscle)	Color & visible fat	Reflectance and transmittance	PLS-DA	R^2^p = 0.70–0.75 RMSEP ≤ 0.1	[59]
NIR	Frozen pork	TVB-N content	Reflectance	PLSR	R^2^p = 0.9669 RMSEP = 2.51 RPD = 5.33	[60]
NIR	Chicken (breast muscle)	Shelf life	Reflectance	PLS-DA	PLS-DA classification accuracy: 91–96% (bench-top), 75–94% (portable)	[61]
FT-IR	Minced beef	Adulteration with rat meat	Attenuated total reflectance	LDA	R^2^p > 0.90 RMSEP = 0.01–0.051	[62]
FT-IR	Minced beef	Spoilage	Attenuated total reflectance	PLSR, SVM, ANN & GP	Correct classification: 90% (PLSR), 93.33% (SVM), 96.67% (ANN), & 90% (GP)	[63]
FT-IR	Beef (longissimus dorsi)	Tenderness	Attenuated total reflectance	PLSR	R^2^p = 0.07–0.38 RMSEP = 0.05–2.11	[64]
Raman	Pork (semimembranosus muscles)	pH, drip loss, shear force	-	PLSR	R^2^cv = 0.22–0.70, RMSECV = 0.09–7.8	[65]
Raman	Beef (Gluteus Medius muscles)	Tenderness	-	PLSR, PLSDA	R^2^p = 0.23–0.33 & RMSEP = 8.5–8.8	[66]
Fluorescence	Beef (longissimus thoracis muscle)	Total fat and fatty acid composition	FFFS and SFS	PLSR	R^2^p ≥ 0.48 & RPD ≥ 1.49	[56]
Fluorescence	Beef (longissimus dorsi and rump steak)	TVB-N, TBARS, and TVC	FFFS	PLS-DA	R^2^p = 0.871–0.95 & RMSEP = 0.054–0.975	[53]
Fluorescence	Minced beef	Adulteration	FFFS	PLSR	R^2^p = 0.86–0.95 RMSEP = 0.56–4.82	[58]
Terahertz Spectroscopy	Chicken (breast muscle)	CCH and TCH	Transmittance	PLSR	R^2^p = 0.9251–0.9575 & RMSEP = 0.0744–0.128	[67]
Terahertz Spectroscopy	Pork (longissimus muscles)	Freshness	ATR	BP-ANN	R^2^p = 0.84 RMSEP = 0.0989	[44]

Abbreviations: TVB-N, total volatile basic nitrogen; TBARS, thiobarbituric acid reactive substances; TVC, total viable count; CCH, chlortetracycline hydrochloride; TCH, tetracycline hydrochloride; FFFS, front face fluorescence spectroscopy; SFS, synchronous fluorescence spectroscopy; PLS-DA, partial least squares discriminant analysis; PLSR, partial least squares regression; GP, genetic programming; SVM, support vector machine; BP-ANN, back propagation artificial neural network; ATR, attenuated total reflectance; R^2^p coefficient of determination for prediction; R^2^cv coefficient of determination for cross validation; RMSECV, root mean square error of cross validation; RMSEP, root mean square error of prediction; RPD, ratio of performance to deviation.

**Table 2:** Overview of commonly used evaluation metrics in machine learning and deep learning models.

Metric (Abbrev.)	Interpretation	Ranges
Coefficient of determination (R^2^)	Indicates proportion of variance explained by the model. A higher R^2^ (closer to 1) is better.	Typical 0–1. High R^2^cv with a lower R^2^p may indicate overfitting.
Root mean square error (RMSEP, RMSECV)	Measures average prediction error in original units. Lower is better.	No fixed good value, but it depends on units.
Residual predictive deviation (RPD)	Higher RPD indicates better predictive ability.	Rough guidelines indicate that RPD <1.5 (poor), 1.5–2 (approximation), 2–3 (moderate), >3 (good).
Accuracy	Overall fraction correct (both classes). Higher is better.	Ranges 0–1. Not reliable if classes are imbalanced.
Sensitivity (recall)	True positive rate: fraction of actual positives correctly identified. Higher is better.	Ranges 0–1. Can be misleading if the data is imbalanced.
F1 Score	Harmonic mean of precision and recall. High F1 means both are high.	Ranges 0–1. Does not consider true negatives. Good when classes are imbalanced, but focus on positive class performance.

### 3.3. Imaging Techniques

#### 3.3.1. Hyperspectral Imaging (HSI)

**Figure 6:**
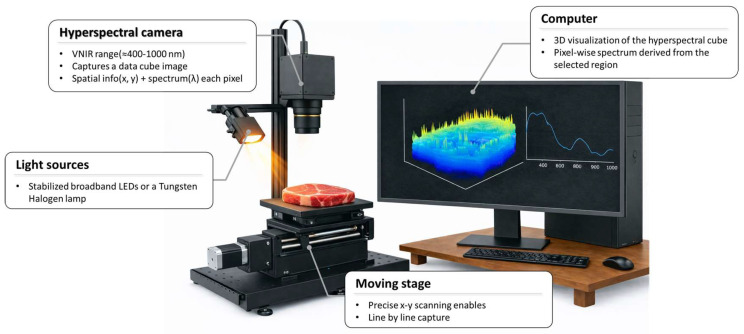
Main benchtop hyperspectral imaging system components.

Hyperspectral imaging (HSI), originally developed for remote sensing in the 1970s–1980s [68], was later adapted for food quality assessment in the late 2000s. Its core output is a three-dimensional hypercube integrating spatial and spectral information, where the x-y axes represent spatial dimensions and the spectral axis (λ) captures wavelength-dependent light intensity, such that each pixel contains a full spectrum at a specific location [69]. A typical HSI system (Figure 6) comprises a light source, hyperspectral camera, objective lens, conveyor-mounted stage, and a processing computer [70]. Hyperspectral images can be acquired using four modes: line scanning, which captures spectral data along a single line; band sequential scanning, where the full area is scanned across wavelengths; point scanning, which records spectra at individual points; and snapshot mode, which captures the full hypercube in a single exposure [71,72,73].

HSI demonstrates strong performance for proximate composition and visible quality traits. Barbin et al. [74] reported R^2^p values of 0.88 (protein), 0.91 (moisture), and 0.93 (fat), with RPD up to 4.0 for fat prediction. Yuan et al. [75] quantified metmyoglobin in sheep longissimus dorsi, identifying key absorption features at 970 nm (moisture-related O-H) and 540–566 nm (respiratory pigments). Freshness and aging classification are also robust. Crichton et al. [76] achieved CCR values of 92–93% for fresh, frozen-thawed, and matured beef, while Haughey et al. [77] reported 74–100% external accuracy for discriminating wet-aged steaks. Additionally, structural abnormalities are effectively detected. The authors of [78] predicted intramuscular fat and pH across species with R^2^p = 0.86–0.89 and RPD values of 2.88 (IMF) and 3.04 (pH). Kim et al. [79] further classified vaccine-induced abscesses in pork (SWIR 1000–2500 nm) with 97–99% accuracy, linked to N–H, O–H, C–H, and collagen features. However, performance declines for attributes lacking distinct spectral signatures. Ye et al. [80] reported only R^2^p = 0.6833 for TVC in chicken, and León-Ecay et al. [81] achieved 67.14% accuracy for tenderness classification, reflecting the complexity of microbial and mechanical traits. Other studies in which HSI was applied can be found in Table 3.

Overall, HSI provides rich spectral detail but faces significant deployment limitations. The generation of large hyperspectral datasets creates heavy computational and storage demands, slowing processing and limiting real-time applications. High equipment costs, complex calibration requirements, and sensitivity to lighting and positioning further reduce robustness in industrial environments. Moreover, its shallow penetration depth restricts analysis to surface layers, preventing internal assessment [68,82]. These constraints translate into reduced throughput and scalability, confining HSI largely to controlled or offline settings rather than high-speed production lines. Thus, HSI is a powerful but resource-intensive technique best suited for detailed compositional mapping. It excels in identifying chemical attributes such as moisture, fat, and pigments, particularly in heterogeneous products. However, its practicality in industry is limited by cost and processing demands.

#### 3.3.2. Multispectral Imaging (MSI)

**Figure 7:**
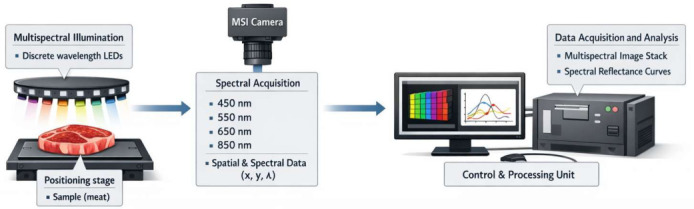
Schematic of the main components of a Multispectral Imaging System.

Multispectral Imaging (MSI), similarly to HSI, integrates spatial and spectral information but captures fewer discrete, non-contiguous bands (typically 3–15), reducing data volume and computational demand [70,83,84]. This streamlined structure enables faster processing and supports real-time industrial applications. However, the limited spectral coverage may overlook subtle features associated with specific quality attributes [85,86]. MSI generates data cubes in which each pixel contains reflectance values at selected wavelengths, commonly within the VIS, NIR, MIR, or FIR regions [87,88,89]. Figure 7 illustrates the components of a typical multispectral imaging system.

In meat quality assessment, MSI demonstrates strong but application-dependent performance. Tsakanikas et al. [90] used MSI with SVR to monitor spoilage in packaged beef, achieving R^2^p = 0.98 for microbial prediction and 80.8–89.2% classification accuracy across storage conditions. Aït-Kaddour et al. [91] reported high predictive accuracy for total collagen content in beef (R^2^p = 0.99; RMSEP = 0.02 mg OH-pro × g^−1^ DM) using artificial neural networks. MSI has also supported beef cut classification, with A. Li et al. [92] achieving accuracy above 90% using optimized linear discriminant analysis and multimodal feature fusion. More recently, Kolosov et al. [93] demonstrated portable MSI systems integrated with deep convolutional neural networks for microbiological quality estimation, underscoring the potential for real-time deployment. Additional applications are summarized in Table 3.

MSI reduces data complexity by focusing on selected wavelengths, improving speed, and simplifying analysis compared to HSI. However, this reduction in spectral information limits sensitivity to subtle chemical differences, lowering accuracy for complex or weakly defined traits. Similar to HSI, MSI is restricted to surface measurements and remains sensitive to environmental conditions such as lighting and positioning. While less computationally demanding, it still requires careful calibration, and its performance depends heavily on appropriate wavelength selection for specific applications.

The authors of this review consider MSI a practical compromise between performance and deployment capability. It is more suitable for real-time industrial use, particularly for targeted quality attributes like color, fat, or moisture. Although less precise than HSI, its lower cost and faster processing make it attractive for inline grading tasks. MSI is best applied where key spectral markers are known, offering a balance between efficiency and analytical capability.

#### 3.3.3. Thermal Imaging

Thermal (infrared) imaging measures the surface temperature and heat-transfer behavior of meat by capturing emitted long-wave IR radiation, which depends on temperature and emissivity [94]. In meat applications, active thermography is commonly used, where controlled heating or cooling is applied, and the transient thermal response is recorded. Variations in thermal conductivity, heat capacity, and emissivity generate image contrast [76,95]. Unlike spectroscopic techniques, thermal imaging reflects physical heat-flow properties rather than molecular composition.

This approach is effective for detecting structural heterogeneity and adulteration. The authors of [96] applied controlled heating to minced mutton and used CNN-based analysis of thermal videos to detect pork adulteration, achieving 99.99% accuracy and R^2^p = 0.993 (RMSEP = 0.025 fraction). Differences in thermal conductivity also allow discrimination between fat and lean tissues, supporting marbling visualization and detection of structural defects. Although microbial metabolism may slightly elevate surface temperature [95], the signal is weak and not sufficiently specific for reliable microbial quantification. Overall, thermal imaging is most suitable for identifying hidden mixtures or anomalies that alter heat-flow patterns. Table 3 contains more studies utilizing thermal imaging.

Thermal imaging is limited by its indirect measurement of meat properties, relying on heat patterns rather than chemical composition. Its sensitivity to environmental factors such as ambient temperature, airflow, and surface moisture reduces reliability and consistency [76,97]. Detecting subtle quality differences is challenging, and active thermography requires controlled heating and timing, slowing operation. These factors hinder deployment in fast-moving industrial lines, where maintaining stable conditions is difficult, and throughput is critical.

Consequently, thermal imaging is positioned as a specialized rather than a general-purpose tool, performing well in identifying structural anomalies, adulteration, or temperature-driven defects under controlled conditions. However, it is not well-suited for broad quality assessment or continuous inline inspection, and its value is limited to targeted, intermittent checks.

#### 3.3.4. Ultrasound Imaging

Ultrasound imaging employs high-frequency acoustic waves (2–10 MHz) to visualize tissue interfaces based on differences in acoustic impedance [98]. In meat and livestock applications, it provides a non-invasive assessment of carcass geometry, distinguishing muscle, subcutaneous fat, and bone. Echo signals generate grayscale B-mode images, enabling structural evaluation. As a density-based technique, ultrasound measures physical traits but does not provide direct chemical information.

Ultrasound is particularly effective for assessing fat deposition and muscle dimensions. Automated analysis of live cattle scans achieved R^2^p = 0.87–0.88 (RMSEP = 7.6–8.7) for backfat thickness. However, estimation of intramuscular fat (IMF%) and marbling was only moderate (R^2^cv = 0.37; RMSECV = 1.31) [99]. In the same study, neural network-based image recognition was applied to estimate beef marbling standard (BMS) scores in live cattle, reporting a correlation coefficient of 0.70. Ultrasound has also been used to predict carcass yield traits, including lean percentage and muscle area, with R^2^cv = 0.84 under calibrated conditions [100]. Additionally, commercial systems such as AutoFOM and UltraFOM are applied in breeding and abattoir settings for fat grading [101]. Table 3 contains additional applications of ultrasound imaging.

While ultrasound offers deep penetration, it requires direct contact and coupling media, making automation difficult in high-throughput environments. Furthermore, measurement accuracy is highly operator dependent, with variability introduced by positioning, pressure, and sample conditions [1]. External factors such as movement or surface irregularities further affect reliability. These challenges limit scalability and consistency, particularly in fast-paced industrial processing settings. Ultimately, ultrasound is a valuable tool for structural assessment and is widely used for predicting fat thickness, muscle depth, and carcass traits. While it cannot provide a detailed chemical composition, its ability to assess internal structures makes it indispensable for specific grading tasks.

#### 3.3.5. X-Ray Imaging

X-ray imaging enables internal evaluation of meat quality and safety by measuring differential attenuation of X-ray photons through tissues of varying density and composition [102]. The two principal techniques are computed tomography (CT) and dual-energy X-ray absorptiometry (DEXA). CT generates cross-sectional and three-dimensional images, allowing accurate quantification of intramuscular fat (IMF) and marbling. Clelland et al. [102] reported a correlation coefficient of 0.67 for CT-based IMF prediction in Texel lamb loin, while later studies demonstrated stronger correlations (R^2^p = 0.80–0.97) with chemical fat analysis, supporting CT as a reference method for nondestructive IMF assessment [103]. DEXA differentiates tissues using two X-ray energy levels, enabling compositional analysis and foreign body detection. Andriiashen et al. [104] achieved 95% accuracy in detecting fan bones in chicken fillets, with improved reliability after thickness correction. Nunes et al. [105] further demonstrated IMF prediction in beef longissimus steaks with R^2^p values above 0.75. Additional applications are summarized in Table 3.

Despite strong analytical capability, industrial adoption is constrained. CT systems are large, stationary installations requiring radiation safety controls and minimal sample movement, limiting use to research or fixed grading stations. DEXA can be integrated into processing lines; however, it is often restricted to smaller cuts. Portable systems remain uncommon due to shielding requirements. Additionally, temperature-dependent attenuation necessitates standardized scanning conditions [1]. Thus, although X-ray and CT provide robust internal compositional insight, practical application remains largely confined to laboratory or fixed-line environments.

In summary, X-ray imaging is a gold standard for internal analysis and safety inspection. It is highly effective for detecting foreign objects and quantifying internal composition, making it valuable in research and specialized applications. However, due to cost and operational constraints, its use is typically limited to offline analysis or specific safety checkpoints. It is best deployed where internal visualization is critical and throughput demands are manageable.

**Table 3:** Summary of Imaging applications in meat quality and safety.

Imaging Technique	Meat Type	Quality Attribute	Analytical Method	Key Wavelengths ^1^	Model Performance	Reference
HSI (400–1000 nm)	Sheep	Myoglobin content	PLSR & LSSVM	DeoMb: 473, 512, 530, 761, 766, 780, 843, 943, 958 nm MbO2: 506, 530, 569, 574, 636, 665, 775, 780, 795, 819, 857, 948 nm MetMb: 410, 454, 473, 506, 521, 530, 579, 646, 756, 790, 819, 948 nm	R^2^p = 0.725–0.901 & RMSEP = 1.157–3.942	[106]
HSI (548–1701 nm)	Beef, lamb, & venison	IMF & pH	PLSR & DCNN	_	R^2^p = 0.75–0.89 & RPD = 2.01–3.04	[78]
HSI (400–1000 nm Vis-NIR) & (1116–1670 nm SWIR)	Minced lamb, beef, & chicken	Adulteration with pork	PLSR, SVM-R, & ANN-MLP	_	R^2^p = 0.02–0.99 & RMSEP = 0.04–0.34	[107]
MSI (405–970 nm)	Beef	Water injection detection	PLSR	450, 470, 435, 570, 780 nm	R^2^p = 0.923–0.946 & SEP = 0.618–0.645	[108]
MSI (405–970 nm)	Beef fillets	TVC	MLP, SVM, PLSR, WNN, & CAGFINN	405, 435, 450, 470, 505, 525, 570, 590, 630, 645, 660, 700, 850, 870, 910, 940, 970 nm	R^2^p = 0.385–0.978 RMSEP= 0.255–1.343, RPD = 1.363–7.17	[109]
X-ray (DEXA)	Beef carcass	Fat	PLSR	_	R^2^p = 0.74–0.99 & RMSEP = 0.012–0.420	[110]
X-ray	Chicken breasts	Foreign matter detection	Active contour segmentation algorithm	_	Accuracy = 95% F1 Score = 74%	[104]
Ultrasound	Pork belly layers	Grading	ResNet models (18, 50, 101, & 152) VGG models (16 & 19) AlexNet	_	Accuracy = 0.902–0.962, F1 Score = 0.895–0.958	[111]
Thermal imaging (7.5–14 μm)	Pork	Adulteration	Multilayer CNN	_	R^2^p = 0.9933 RPD = 12.239 RMSEP = 0.0252	[96]
Thermal imaging (7.5–14 μm)	Mutton	Adulteration	ResNet-18	_	R^2^p = 0.9717, RMSEP = 0.0238, & RPD = 7.8696	[112]

Abbreviations: TVC, total viable count; IMF, intramuscular fat; DeoMb, deoxymyoglobin; MbO_2_, oxymyoglobin; MetMb, metmyoglobin; PLSR, partial least squares regression; LSSVM, Least Squares support vector machine; DCNN, deep convolutional neural networks; ANN-MLP, artificial neural network—multilayer perceptron; SVM-R, support vector machine regression; SVM, support vector machine; WNN, wavelet neural network; MLP, multilayer perceptron; CAGFINN, Clustering-based asymmetric Gaussian fuzzy inference neural network; ResNet; residual network, VGG; visual geometry group network, AlexNet; deep CNN architecture (named after Alex Krizhevsky), CNN, convolutional neural network; R^2^p, coefficient of determination (prediction set), RMSEP; root mean square error of prediction; SEP, standard error of prediction; RPD, ratio of performance to deviation. ^1^ The selected key wavebands represent molecular assignments that correspond to fundamental chemical components and their variations in meat products. Water-related absorptions around 940–970 nm, protein-related N-H bonds at 1460–1570 nm, fat-related C-H bonds at 1100–1400 nm, and myoglobin-related absorptions in the visible region (430–760 nm).

Refer to Table 2 for the interpretation of the model performance based on the evaluation metrics used.

### 3.4. Multimodal Sensor Fusion Systems

Multimodal systems integrate sensors that probe different physical or chemical properties of meat, combining complementary information through data, feature, or decision level fusion. For example, Huang et al. [9] fused NIR, RGB imaging, and e-nose signals to predict pork TVB-N, while C. Liu et al. [113] combined HSI and e-nose sensors for assessing lamb TVB-N. Each modality contributes distinct information: imaging captures appearance and composition, e-nose detects volatile compounds, and thermal imaging reflects heat-transfer patterns. Fusion leverages these diverse signals to enhance predictive robustness.

Such systems perform particularly well for freshness, spoilage, and adulteration attributes associated with concurrent chemical and volatile changes. J. Cheng et al. [114] fused hyperspectral and e-nose data to predict pork water content (R^2^p = 0.953; RMSEP = 0.387 g/g), while C. Liu et al. [113] reported R^2^p = 0.920 (RMSEP = 3.04 mg/100 g) for lamb TVB-N. Kodogiannis and Alshejari [115] combined MSI with an e-nose to predict spoilage bacteria, achieving R^2^p above 0.98 (RPD above 5.4) for Pseudomonas and Brochothrix, substantially outperforming individual sensors. Similarly, S. Wang et al. [112] fused thermal and RGB imaging to detect species adulteration, reaching 99.3% classification accuracy and R^2^p = 0.972 (RMSEP = 0.0238; RPD = 7.87) for pork proportion prediction. Overall, fusion systems consistently enhance prediction where multiple physical and biochemical signals evolve simultaneously.

Despite superior accuracy compared with single-sensor approaches, most multimodal systems remain laboratory-based. Integrating multiple sensors increases cost, system complexity, and calibration demands. Each modality requires individual calibration, followed by alignment and fusion of outputs. As the authors of [116] emphasize, constructing and harmonizing spectral databases across modalities is resource-intensive. Calibration transfer challenges already present in single-sensor systems are amplified in multimodal frameworks, limiting large-scale industrial deployment.

### 3.5. Electronic Nose (E-Nose) Systems

Electronic noses (e-noses) are sensor arrays (metal-oxide, polymeric, or colorimetric) coupled with pattern-recognition algorithms to detect volatile organic compounds (VOCs) in meat headspace, generating multidimensional odor fingerprints [117,118]. They mimic olfaction by measuring gaseous byproducts of microbial metabolism and lipid oxidation, but do not directly assess water, fat, or protein composition [119].

E-noses are particularly effective for freshness, spoilage, and odor-related adulteration. Microbial activity releases ammonia and amines; for example, Damdam et al. [119] reported spoilage-related increases in CO_2_ (552–4751 ppm), NH_3_ (6–8 ppm), and C_2_H_4_ (18.4–21.1 ppm) in beef headspace. W. Jia et al. [120] achieved 95% accuracy for spoiled vs. fresh beef using SVM with 10-fold cross-validation. E-noses also detect boar taint with 96.5% sensitivity and 95.3% specificity [121], and low-level pork adulteration in beef with 98–100% accuracy (R^2^p = 0.85–0.99; RMSEP = 0.147–0.85) [122,123]. A 1D-CNN plus Random Forest model quantified pork-in-beef down to 5% (R^2^p = 0.9852; RMSEP = 2.430) [124], while Putri et al. [123] reported accuracies above 90% for classifying beef, pork, and chicken meat floss.

However, e-noses cannot directly quantify bulk composition (water, intramuscular fat, protein, collagen) or physical traits such as pH, tenderness, and texture, as these lack distinct VOC signatures. Although advantages include rapid response, portability, and low cost [125], industrial application is constrained by environmental sensitivity. Stable headspace sampling and sensor equilibration are difficult in processing environments. Humidity, temperature fluctuations, surface residues, and baseline drift affect signal stability, necessitating frequent recalibration or sensor replacement [120]. Consequently, while highly effective for odor-driven attributes, e-noses remain limited for comprehensive meat quality assessment. Table 4 below summarizes these sensing technologies by best-fit attributes, throughput, cost, and readiness level, providing a comparative basis for their integration into industrial meat quality and safety assessment.

## 4. Data Analysis Techniques

Conventional image analysis for meat quality and safety evaluation relied primarily on color space analysis (RGB, HSV/HSB, HSL, and CIELAB) [3,126,127] and texture-based approaches such as gray-level co-occurrence matrix (GLCM) features [128] and wavelet transform decomposition [129,130] to assess appearance, composition, and surface properties. While effective for targeted applications, such as PSE pork and DFD beef detection, these conventional methods were constrained by manual feature selection bias, limited capacity to model nonlinear relationships, and poor generalization across meat types, collectively restricting their scalability and practical deployment.

Artificial intelligence has emerged as a transformative tool in meat quality and safety assessment, addressing these fundamental limitations through automated feature extraction and predictive modeling. Primary applications include regression (for predicting composition, pH, and microbial load), classification (for freshness grading, species authentication, and detecting adulteration), segmentation (for marbling and defect identification), and time-series forecasting for shelf-life estimation. The following section reviews the principal machine learning and deep learning architectures employed in the evaluation of meat quality and safety, highlighting their methodological foundations, performance characteristics, and applicability across key industrial endpoints.

### 4.1. Machine Learning

#### 4.1.1. Linear and Latent Variable Models

Partial least squares regression and discriminant analysis (PLSR/PLSDA) remain widely used for predicting meat composition from spectroscopic data. When properly calibrated, these models typically achieve high coefficients of determination (R^2^) and low prediction errors (RMSEP below 1%) for major constituents. For example, portable VIS-NIR PLSR models have predicted pork quality attributes such as color and water activity with R^2^p values of 0.7–0.9 [22]. Similarly, in Fourier-transform IR (FT-IR) and Raman applications, PLSR demonstrates good performance for composition analysis and adulteration detection [131,132]. For classification, Hu et al. [133] optimized PLSDA to achieve 99% accuracy in distinguishing pure from adulterated beef and enabled quantification of offal types with R^2^p above 0.81. These findings confirm that latent-variable methods perform well in chemically complex matrices when systematic variation is adequately captured.

A principal strength of these approaches is interpretability. Model loadings and variable importance in projection (VIP) scores identify influential wavelengths associated with spoilage markers or pigments [131,134], providing mechanistic insight and regulatory transparency. However, performance is highly dependent on preprocessing and the quality of the spectral data. Baseline correction and multicollinearity management are often required to stabilize models; otherwise, overfitting may occur despite cross-validation. PLS-DA is similarly vulnerable to optimistic accuracy estimates, with performance often declining when applied to independent batches. Overall, PLSR/PLSDA are preferred for tabular spectral data when sample sizes are limited and interpretability/transparency is needed but should be supplemented by other models if nonlinearity is evident or if performance needs to improve beyond the latent-variable limits.

#### 4.1.2. Margin-Based and Kernel Methods

Support vector machines (SVMs) and support vector regression (SVR) are widely applied to spectral and e-nose datasets, particularly in high-dimensional, low-sample scenarios. These margin-based models often demonstrate strong generalization. For instance, in small-cut mutton chemical prediction, SVM maintained robust performance in high-dimensional spaces without degradation [135]. In NIR-based species and adulteration detection, SVM classification accuracy (90–95%) is typically comparable to or slightly higher than PLSDA [136]. Their flexibility stems from nonlinear kernel functions, which model complex spectral relationships without requiring deep architectures, while regularization enhances stability in limited-data contexts [135,137].

Despite these advantages, scalability remains a limitation. Training time increases substantially with dataset size, restricting the application to small datasets. Interpretability is also reduced, as predictions are derived from weighted support vectors in kernel space rather than explicit variable contributions. Although Explainable AI (XAI) tools such as SHapley Additive exPlanations (SHAP) or Local Interpretable Model-agnostic Explanations (LIME) can provide post hoc explanations, PLS-based models are often preferred when mechanistic insight is required. In summary, SVM/SVR are strong choices for moderate-sized spectral problems with nonlinear patterns, serving as useful benchmarks that often outperform PLSR on complex tasks but require careful tuning and more computing.

#### 4.1.3. Tree-Based and Ensemble Learning

Ensemble tree-based methods, including Random Forest (RF), ExtraTrees, and gradient boosting algorithms such as XGBoost and LightGBM, remain underutilized in meat sensing despite strong performance potential. These models handle mixed feature scales, capture nonlinear relationships, and provide feature-importance metrics, making them well-suited to complex spectral and e-nose datasets.

Random Forest, which aggregates decorrelated decision trees via bootstrap sampling and random feature selection, is notably resistant to overfitting in high-dimensional settings. In poultry applications, RF achieved 99% precision for detecting bacterial contamination using e-nose data, slightly outperforming SVM (98.6%) [138]. Similarly, among five machine learning models evaluated for bacterial detection in meat, RF delivered the highest accuracy (97.46%) [139].

Gradient boosting approaches further enhance predictive power by sequentially correcting predecessor errors. Guo [140] reported accuracies above 98.90% for beef quality assessment using an optimized XGBoost model with e-nose data, reflecting its ability to model complex feature interactions beyond linear methods such as PLSR. Built-in regularization and pruning mechanisms improve overfitting control relative to RF’s bagging strategy. LightGBM and related variants offer computational efficiencies through histogram-based splitting, though applications in meat science remain limited. For example, LightGBM achieved 93.7% accuracy for freshness classification compared to 88.9% for SVM [141].

More advanced ensemble strategies, such as stacking, are emerging but remain rare in this domain. J. Zhang et al. [142] demonstrated that a symmetric stacking ensemble learning (SSEL) network outperformed conventional models, achieving R^2^p values above 0.91 and 0.88 with RMSEP below 2.28 and 1.83 for TVB-N and pH prediction in lamb meat. These findings suggest that ensemble integration can enhance robustness beyond single-model approaches. However, stacking requires careful validation to prevent information leakage and demands additional data for meta-learner training, limiting routine adoption.

Despite consistent performance advantages, ensemble methods remain constrained by practical considerations. They generally require large, well-annotated datasets, whereas industrial meat datasets are often small, imbalanced, and costly to label. Computational burden, model complexity, and reduced interpretability compared to simpler algorithms further restrict widespread implementation. Generally, tree ensembles are recommended when datasets are sufficiently large and diverse, accepting reduced interpretability for improved accuracy.

#### 4.1.4. Distance-Based and Probabilistic Models

K-nearest neighbors (KNN) and Naïve Bayes (NB) typically serve as baselines. KNN classifies a sample by voting among its nearest spectral neighbors. It is easy to implement but suffers in high dimensions and can be slow at inference. On the other hand, NB computes likelihoods assuming feature independence. It is surprisingly effective when the independence assumption holds approximately but generally underperforms relative to more sophisticated models. Few studies achieve top performance with KNN or NB in meat quality tasks, but they provide performance checks [143,144]. If a complex model fails to outperform KNN/NB, it hints at data quality issues. We will emphasize that neither KNN nor NB is generally recommended as a final solution for meat spectroscopy, except as a simple benchmark or when extremely fast decisions are needed.

#### 4.1.5. Shallow Artificial Neural Networks

A multilayer perceptron (MLP), while capable of modeling moderate nonlinearities, usually underdelivers when data are scarce. Meat spectral datasets are typically too small to train MLPs without severe overfitting, and well-tuned linear models often surpass them under these conditions. Panagou et al. [145] noted that even when an MLP was trained on FTIR spoilage data, PLSR still achieved higher classification accuracy (95% vs. 74%) and better generalization. In other words, with limited samples, MLPs rarely offer a clear advantage. They occupy a position between linear models and deep nets. Unless a spectral problem is large-scale and highly nonlinear, it is recommended to apply PLSR/SVM. Conversely, when truly large labeled datasets become available, researchers tend to favor convolutional neural networks (CNNs) over MLPs due to architectural efficiency and feature-learning capability [146].

### 4.2. Deep Learning

#### 4.2.1. Convolutional Neural Networks

**Figure 8:**
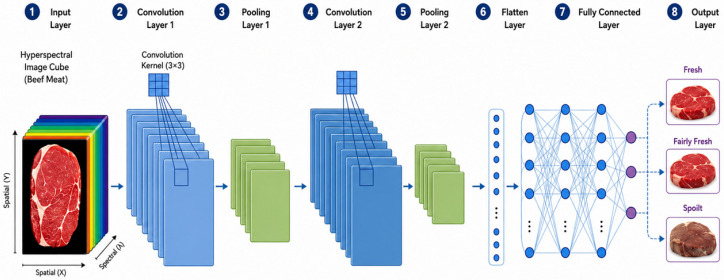
Schematic illustration of a convolutional neural network architecture.

Convolutional neural networks (CNNs) have transformed image-based meat quality assessment by automatically learning hierarchical spatial features from raw pixels. By convolving learnable filters over the input, CNNs can automatically capture textures, edges, and complex spatial patterns without manual feature engineering. A typical CNN comprises an input layer, alternating convolutional and pooling layers, and fully connected output layers (Figure 8). In meat applications, CNNs have achieved outstanding results. For example, a 1D spectral CNN achieved 94.4% accuracy in ground beef adulteration detection [147]. Similarly, a Vision Transformer combined with CNN-based feature extraction enabled near-perfect foreign-object detection in pork belly [148]. Their strength is especially clear when the input has rich spatial structure or when combining spectral and spatial information (3D-CNNs for HSI cubes). However, CNNs are justified only when those data structures and volumes are present. They demand far more training data (often thousands of labeled images) and computational resources (GPU training and inference) than classical methods. Without such data, CNNs overfit easily. As noted above, large hyperspectral cubes lead to the curse of dimensionality, and insufficient samples can degrade performance. Thus, CNNs are recommended for meat quality tasks involving 2D/3D imagery where enough examples exist to train deep filters, but one should always weigh their impressive accuracy against the practical costs, such as model complexity, inference latency, and hardware needs.

#### 4.2.2. Attention Mechanisms and Transfer Models

A limitation of purely convolutional models is their local receptive field. A single kernel spans only a few contiguous bands, so long-range relationships across the spectrum are captured only indirectly. Attention mechanisms address this by weighting informative spectral regions dynamically. Cai et al. [149] built a pyramid attention feature fusion model (PAFFM) by integrating convolution, an attention mechanism, and a pyramid structure, to predict total volatile basic nitrogen (TVB-N) and total viable count (TVC) in chicken breasts, explicitly mitigating information loss across long spectral distances.

Additionally, many meat imaging studies now use transfer learning to mitigate limited data. A CNN pretrained on large natural image datasets is fine-tuned on the meat image task. This typically achieves high accuracy with only a few hundred labeled images, because early convolutional layers have learned generic texture and color features that often translate to meat surfaces. For example, fine-tuned ResNet-50 or VGG-19 models have reported 98.8% accuracy on pork freshness classification with a few hundred RGB images [150]. It is worth noting that transfer learning is especially effective for visible RGB images of meat (color, texture, marbling), but it is less clear for hyperspectral data whose spectral patterns differ from natural images. Some works even suggest domain-specific pretraining for HSI using CNNs trained on other spectral data [151,152]. Consequently, transfer learning is an efficient strategy when only moderate-sized image datasets are available, providing much of the accuracy of a full CNN with far less data.

#### 4.2.3. Temporal Models

When the data are time sequences rather than static spectra or images, recurrent neural networks and their variants become relevant. For example, electronic-nose sensors collecting odor signals over time during meat spoilage can be fed to LSTM networks. These models capture time correlations such as rising spoilage volatiles to forecast near-term freshness. X. Li et al. [153] demonstrated an LSTM-based system achieving R^2^ = 0.950 (RMSE = 0.097) for 1-h-ahead TVC prediction and R^2^p above 0.859 (RMSEP = 0.147) for 9-h-ahead forecasts, substantially outperforming static models. GRUs and temporal convolutional nets have also been explored for shelf-life forecasting. It should be noted that temporal models shift assessment toward predictive control, which is valuable for IoT quality monitoring. However, the trade-offs are the need for well-aligned time-series data, potentially high computational load, and diminished interpretability. Their use is still relatively rare in meat quality, thus validation on diverse products is encouraged if adopted.

#### 4.2.4. Autoencoders and Representation Learning

Autoencoders learn compressed, unsupervised embeddings of spectral or image data. In meat sensing, they have mainly been applied to anomaly or outlier detection. For instance, an autoencoder trained on “normal” hyperspectral pork belly images can flag foreign objects as deviations in the latent space. In fact, Lee et al. [154] demonstrated that an optimized autoencoder significantly outperformed earlier CNN approaches for hyperspectral foreign-object detection. Notably, autoencoders can also reduce dimensionality before classification, but unlike PCA, the learned features lack simple physical meaning. Therefore, autoencoder-based methods offer powerful unsupervised feature learning or anomaly detection, but at the cost of full transparency. They overfit if the data are small and are mostly exploratory, thus have not yet become routine in meat quality.

#### 4.2.5. Generative Models and Data Augmentation

Generative models are emerging as a data-augmentation and semi-supervised tool. For example, Generative Adversarial Networks (GANs) have been used to synthesize realistic meat images or spectra. One study on poultry HSI used a GAN with a 1D U-Net discriminator to generate contaminant-free spectra, achieving 100% precision and over 96.5% recall in detecting 30 contaminant types [155]. Diffusion models are beginning to appear for spectral augmentation. Generative methods can boost training data diversity when real samples are scarce, but they introduce new risks. If the GAN fails to capture rare real-world variability, downstream models may become overly optimistic [156]. Accordingly, the use of synthetic data requires careful validation using real-world samples to confirm that observed performance improvements are substantive. These considerations are closely linked to broader issues of generalization, reproducibility, and validation that extend beyond model architecture.

#### 4.2.6. Deep Learning Architectures for Spectral and Imaging Data

Convolutional neural networks (CNNs) have become central to spectral analysis. One-dimensional CNNs navigate the spectral sequence with learnable kernels to extract local features such as peak shapes, slopes, and inflection points, with little manual preprocessing or wavelength selection. For meat specifically, hybrid architectures that couple convolutional feature extraction with recurrent layers are effective. Yan et al. [157] combined a CNN with a bidirectional long short-term memory network (CNN-Bi-LSTM) to predict linoleic acid in mixed red meat (Tan lamb, beef, and pork) from hyperspectral images, capturing sequential dependencies along the spectral axis in both directions. They further embedded Bayesian optimization, via a Gaussian-process acquisition function, to tune hyperparameters and limit overfitting, raising the prediction coefficient of determination from 0.889 for the base CNN-Bi-LSTM to 0.909 for the Bayesian-optimized model, an early example of uncertainty-aware model selection in meat spectroscopy.

### 4.3. Multimodal Fusion

No single sensor fully characterizes the coupled chemical, microbial, and structural changes of meat spoilage, which motivates multimodal fusion. Liu et al. [113] fused electronic-nose (e-nose) signals with hyperspectral imaging through an input-modified CNN to predict TVB-N in mutton, combining screened gas-sensor responses with effective-wavelength and image features to reach a prediction-set correlation coefficient of 0.920 and a ratio of performance to deviation of 3.59. More recent work replaces fixed fusion rules with learned, attention-based integration. Cheng et al. [158] proposed a Hybrid Fusion Attention Network (HFA-Net) to combine fluorescence hyperspectral imaging with e-nose data for pork freshness, explicitly designed to overcome the dimensionality burden of early fusion and the loss of cross-modal interactions in late fusion. Fusion of complementary spectral ranges is likewise beneficial. The dual-range (visible near-infrared plus short-wave near-infrared) attention model of Cai et al. [149] exploits both regions within a single end-to-end network for chicken.

## 5. Challenges and Limitations

### 5.1. Instrumental Limitations

Despite strong analytical performance, the reviewed nondestructive techniques face instrumental barriers to large-scale industrial deployment, primarily arising from sensor design, environmental sensitivity, hardware durability, and integration with high-throughput lines.

Optical systems require stable illumination and fixed sensor sample geometry. Fluctuations in light intensity, source spectral stability, temperature, humidity, and mechanical vibration degrade the signal-to-noise ratio and compromise accuracy and repeatability. High-resolution imaging platforms further demand controlled acquisition distances and prolonged scanning, conflicting with the throughput of automated production. Electronic nose (e-nose) systems add sensor drift, poor reproducibility, cross-sensitivity to non-target volatiles, and limited operational lifespan, with long-term calibration stability the principal obstacle to sustained operation.

Cost and scalability compound these constraints. Laboratory-grade instruments are typically expensive, bulky, and insufficiently rugged for conveyor environments. Designing compact, durable, and economically viable sensors without sacrificing analytical resolution remains a central engineering challenge. Table 4 summarizes these constraints and compares deployment readiness across techniques.

### 5.2. Computational/Model Limitations

Nondestructive sensing generates high-dimensional datasets that challenge conventional analytics. Tree-based ensembles and convolutional neural networks (CNNs) frequently outperform linear models, but at the cost of interpretability and heightened overfitting risk. Deep CNNs and multilayer ANNs also require large labeled datasets and substantial computational resources, straining real-time industrial cost and latency budgets.

Generalization poses a further limitation, as models optimized on laboratory data often degrade across new batches, breeds, suppliers, or processing environments. This domain shift, driven by seasonal variation, raw-material heterogeneity, packaging differences, or environmental change, undermines model stability. Temperature-induced spectral drift is a representative case. Fodor et al. [23] reported that minor thermal fluctuations shift NIR/IR spectral peaks, necessitating compensation or strict thermal control. Robust feature extraction, calibration transfer, and domain adaptation are therefore essential for cross-condition reliability.

Model selection is further complicated by the bias-variance tradeoff. Transparent, reproducible methods such as PLSR and shallow SVMs may underfit complex nonlinear relationships, whereas deep architectures capture subtle patterns but operate as black boxes and can inadvertently learn noise. This limited interpretability, as emphasized in [159] challenges quality-control systems that must justify decisions within HACCP or ISO frameworks. Edge-computing constraints intensify the problem. Real-time inference with deep models on embedded hardware remains difficult without cloud connectivity or dedicated accelerators, raising latency and reliability concerns. IoT and blockchain frameworks can strengthen traceability and data integrity but increase system complexity, and continuous retraining demands revalidation that adds time and cost. High dimensionality, overfitting, calibration transfer, interpretability, and data governance thus remain the principal barriers to moving AI-driven sensing from laboratory prototypes to robust industrial analytics.

### 5.3. Deployment and Operational Challenges

Integrating nondestructive sensors into processing lines introduces logistical and operational barriers beyond the instrumental and computational. Line speed and throughput are primary concerns, and packaging interference further complicates inline use. Film translucency and adhesive layers distort optical and NIR signals, while opening packages for inspection raises cycle time and labor. Jia et al. [160] note that such real-world factors continue to hinder the in-factory application of hyperspectral imaging (HSI).

Slaughterhouse conditions impose stringent engineering demands. Frequent high-pressure washdowns, strict hygiene protocols, and exposure to moisture, heat, vibration, and corrosive atmospheres require ingress protection and durability that many laboratory prototypes lack. Sensor fouling compounds the problem, as accumulated blood or fat on optical interfaces obstructs signal transmission and degrades performance [161].

Supply-chain variability and the absence of standardization present additional obstacles. Facilities that process multiple species, cuts, or formulations typically require extensive retraining to transfer a model between products, yet no universal standards exist for calibrating or reporting nondestructive measurements in meat. This gap complicates data governance. Model ownership, update tracking, cross-plant algorithm sharing, and sensor-data traceability also remain undefined. Although blockchain and secure data platforms have been proposed, most plants lack the IT infrastructure for real-time analytics across supply networks. Adoption economics, finally, divide large processors, which can absorb high initial costs and retrofit lines, from SMEs, for which such investment is often prohibitive absent clear cost-benefit evidence. Where maintenance and lifetime costs outweigh perceived quality-control gains, scalability remains contingent on favorable economics and regulatory harmonization, explaining why many promising laboratory methods stall at the pilot stage.

## 6. Future Research Directions

Translating nondestructive sensing from laboratory prototypes to industrial practice depends on progress across four interlocking fronts: robust sensing hardware, contamination-resistant probe design, miniaturization with edge intelligence, and adaptive model transfer, each underpinned by standardized validation.

Sensing hardware must operate reliably in industrial meat-processing environments, which prioritizes drift-resistant architectures with automated self-calibration and diagnostics. Sealed optical assemblies, thermal stabilization modules, and internal reference standards can compensate for temperature and vibration induced drift [162] directly addressing the instability that undermines measurement on high-throughput lines. Contamination-resistant, self-cleaning probes are equally critical. Solvent and vapor flushing designs proven in pharmaceutical process monitoring, such as the Lighthouse and FDR-825 probes [163], could be adapted to minimize optical fouling and manual cleaning. Multispectral probe heads that co-align near-infrared (NIR), mid-infrared (MIR), Raman, and fluorescence modalities would further capture complementary compositional and structural information from a single measurement location [164].

Miniaturization extends these gains to high-speed lines. Micro-electromechanical systems (MEMS) spectrometers and compact laser or LED arrays enable smaller, lower-cost sensors, while embedding edge computing for on-device preprocessing and inference reduces the bandwidth and latency of cloud-based analytics. Declining sensor costs and improved energy efficiency are already enabling distributed, in-line deployment [165].

Because models optimized under one condition degrade under others, robust calibration transfer and adaptive analytics are essential to preserve performance across instruments, facilities, and processing conditions. Spectral Difference by Wavelength (SDW) strategies, including Direct Standardization (DS) and Piecewise Direct Standardization (PDS), align spectral responses between instruments [166,167] and could be embedded in automated pipelines that calibrate newly deployed sensors from a limited set of standard measurements. Standard-free domain adaptation offers a complementary route. Domain-invariant partial least squares (di-PLS) and Transfer Component Analysis (TCA) align spectral distributions across batches and instruments without dedicated reference samples, while orthogonal projection methods such as External Parameter Orthogonalization (EPO) and Dynamic Orthogonal Projection (DOP) correct environmental perturbations including temperature, humidity, and mechanical motion [168]. Embedding these corrections within adaptive modeling frameworks is critical for stable prediction under continuously changing operating conditions.

Realizing this potential ultimately requires standardized validation aligned with established food-safety systems. Spectral monitoring can be integrated into ISO 22000 and HACCP by linking sensor outputs to defined critical control points and ensuring analytical traceability for regulatory auditing [169]. Such protocols will build industrial confidence and accelerate adoption, shifting meat processing from reactive inspection toward predictive, data-driven quality management, with significant gains in yield optimization, food-safety assurance, and production efficiency.

## 7. Conclusions

In this review, we examined various nondestructive measurement technologies for evaluating meat quality and safety, emphasizing their analytical capabilities, limitations, and readiness for industrial deployment. The review covered major sensing approaches, including CVS, spectroscopic techniques (NIR, FT-IR, Raman, terahertz, and fluorescence), imaging technologies (HSI, MSI, thermal, ultrasound, and X-ray), e-nose systems, and multimodal sensing platforms. Among these, multimodal systems offer clear advantages over single-sensor approaches by integrating complementary information streams, thereby improving prediction accuracy, robustness, and application versatility. However, their practical implementation remains largely confined to laboratory settings due to higher system complexity and implementation costs.

Although nondestructive technologies are steadily advancing toward commercial meat processing environments, adoption remains uneven. CVS and NIR spectroscopy have achieved early industrial use, whereas advanced techniques such as HSI, MSI, Raman spectroscopy, and e-nose systems are still largely at the research or pilot stage. Key barriers include high equipment costs, sensor fouling, calibration transfer challenges, regulatory validation requirements, computational demand, and the need for specialized data analytics. Future progress will depend on cost-effective hardware development, standardized spectral libraries, robust and self-cleaning sensor designs, adaptive machine-learning algorithms, and large-scale cross-plant validation. Ultimately, translating AI-enabled nondestructive sensing from laboratory innovation to routine industrial practice will be critical for achieving scalable, real-time, and data-driven quality and safety assessment across the global meat industry.

## Figures and Tables

**Table 4:** Comparative overview of nondestructive sensing techniques.

Technique	Best-Fit Attributes	Quality /Safety	Throughput	Cost Band	Readiness (TRL)	Implementation Challenges
CVS (computer vision)	Surface color, marbling, PSE/DFD defects, gross freshness	Both (mostly quality)	High (inline, real-time)	Low	High (7–9)	Lighting control required, poor detection of composition (protein, water, microbes), and hidden defects.
NIR spectroscopy	Composition (fat, protein, moisture), TVB-N, color, shelf-life	Both	High (point/inline)	Low–Med	High (7–9)	Limited penetration depth in dense meat, high sensitivity to temperature, humidity, relatively expensive hardware, and calibration transfer complexity.
FT-IR spectroscopy	Adulteration, spoilage, tenderness	Both	Medium	Medium	Medium (5–7)	Very limited depth, water interference, need for strict environmental control, and fairly high instrument cost.
Raman spectroscopy	Targeted composition, tenderness, pH/drip loss	Quality	Low–Med	High	Low–Med (4–6)	Weak Raman signals cause low SNR, fluorescence masking, expensive lasers/detectors, and a need for careful environmental control.
Terahertz spectroscopy	Moisture/water status, freshness, and packaged contamination	Both	Low	Very high	Low (3–4)	High equipment cost, bulky, sample size sensitivity, and shallow penetration depth, as THz is strongly absorbed by water.
Fluorescence spectroscopy	Spoilage markers (TVB-N, TBARS, TVC), fat/fatty acids	Both (safety-leaning)	High	Low–Med	Medium (5–7)	Strong interference from meat autofluorescence, requires high-sensitivity detectors, photobleaching, costly.
HSI (Hyperspectral imaging)	Spatial composition (IMF, pH), myoglobin, adulteration, microbial load	Both	Medium (heavy compute)	High	Medium (5–7)	Very high data volume and processing needs, expensive optical systems, lighting.
MSI (multispectral imaging)	Water/injection detection, TVC, surface quality	Both	Medium–High	Medium	Medium (5–7)	May miss critical spectral features, lower accuracy.
Thermal imaging	Adulteration, surface temperature anomalies	Both	Medium	Medium	Low–Med (4–6)	Sensitive to ambient conditions (drafts, heat sources), requires regular calibration.
X-ray/DEXA/CT	Bulk fat/lean, bone, foreign-body detection	Both (safety for FB)	Medium–High	High	Medium–High (6–8)	Very high capital and maintenance costs, regulatory/safety (radiation shielding) CT requires significant processing time.
Ultrasound	Fat/muscle depth, tenderness, grading	Quality	Medium	Medium-High	Medium (5–7)	Requires a coupling medium, and the signal is affected by fat/water variability.
E-nose	Freshness, spoilage, odor, adulteration, boar taint	Majorly safety	Medium	Low–Med	Medium (5–7)	Sensor drift, humidity effects, limited to odor-active attributes, poor reproducibility, and requires stable airflow.
Multimodal/ sensor fusion	Freshness, spoilage, adulteration (concurrent chemical + volatile change)	Both	Low–Med (integration overhead)	High	Low–Med (4–6)	Complex integration of hardware and data (synchronization, big data analysis), high cost, calibration transfer challenges.

## Data Availability

The original contributions presented in this study are included in the article/Supplementary Materials. Further inquiries can be directed to the corresponding author.

## Supplementary Materials

The following supporting information can be downloaded at: https://www.mdpi.com/article/10.3390/foods15152592/s1, PRISMA 2020 Checklist [18].
