# Supplementary material for: AI-Driven Nondestructive Measurement Technologies for Meat Quality and Safety: A Review
Source: Foods. 2026 Jul 24;15(15):2592. doi: 10.3390/foods15152592 (PMC13465338; doi:10.3390/foods15152592)
Supplement: Supplementary file 1 [file foods-15-02592-s001.zip › foods-4399681-supplementary.pdf]

PRISMA 2020 Checklist

Section and Topic	Item #	Checklist item	Location where item is reported
**TITLE**			
Title	1	Identify the report as a systematic review.	Title page (p.1). The article is identified as a 'Review'
**ABSTRACT**			
Abstract	2	See the PRISMA 2020 for Abstracts checklist.	Abstract (p. 1) states the scope, aims, and key findings of the study.<br>Keywords: meat quality, meat safety, nondestructive techniques, artificial intelligence, industrial deployment
**INTRODUCTION**			
Rationale	3	Describe the rationale for the review in the context of existing knowledge.	Introduction (p. 2-3). States the knowledge gap as existing literature frequently emphasizes sensing techniques, model development, and prediction performances, while comparatively less attention has been given to real-world deployment status and challenges limiting the deployment of Non-destructive technologies, coupled with AI-driven analysis for industrial use.
Objectives	4	Provide an explicit statement of the objective(s) or question(s) the review addresses.	Introduction (p. 2-3). In this review, advances in both sensing modalities and Artificial Intelligence analytical methods were examined with a focus on industrial applicability. Also, a scope roadmap was included in section 1.
**METHODS**			
Eligibility criteria	5	Specify the inclusion and exclusion criteria for the review and how studies were grouped for the syntheses.	Literature Search Strategy, p. 3: search window (Jan 2015-Apr 2026) and screening "against the inclusion criteria"; exclusion categories and counts appear in Figure 2 (p. 4).
Information sources	6	Specify all databases, registers, websites, organisations, reference lists, and other sources searched or consulted to identify studies. Specify the date when each source was last searched or consulted.	Literature Search Strategy, p. 3: Web of Science, ScienceDirect, Google Scholar, and PubMed (microbiological/food-safety records); coverage January 2015-April 2026. Specific search dates were not documented.
Search strategy	7	Present the full search strategies for all databases, registers and websites, including any filters and limits used.	Literature Search Strategy (p. 3): search-term groups (sensing-technology, meat-matrix, quality/safety-attribute, and analytics terms) combined with Boolean AND/OR operators. The specific format used was: (sensing-technology term) AND (meat-matrix terms) AND (quality/safety-attribute terms) AND (analytics terms), each searched one at a time. For example:<br>**Google Scholar (no field tags; supplementary coverage):**<br>meat "machine learning" (near-infrared OR hyperspectral OR "electronic nose") (freshness OR adulteration OR spoilage).<br>**Web of Science (Topic search, TS=):**<br>TS=(("near-infrared" OR NIR OR hyperspectral OR Raman OR terahertz OR THz OR fluorescence OR "computer vision" OR "machine vision" OR "electronic nose" OR e-nose OR multispectral OR ultrasound OR "X-ray" OR "computed tomography" OR CT OR DEXA) AND (meat OR beef OR pork OR poultry OR chicken OR lamb OR mutton) AND (freshness OR "TVB-N" OR "total volatile basic nitrogen" OR "microbial load" OR spoilage OR adulteration OR marbling OR composition OR "fat content" OR "protein content" OR moisture) AND ("machine learning" OR "deep learning" OR chemometrics OR "neural network" OR "partial least squares" OR "support vector" OR "calibration transfer")), Refined by: Publication years 2015-2026; Document type = Article; Language = English.<br>**ScienceDirect (limited Boolean operators per field, run modality-specific searches):**

Section and Topic	Item #	Checklist item	Location where item is reported
			Title-abstract-keywords: ("nondestructive" AND meat AND ("machine learning" OR "deep learning" OR chemometrics) AND (freshness OR spoilage OR adulteration OR composition)); filtered to research articles, 2015–2026. Repeat, substituting each sensing modality (NIR, hyperspectral, Raman, terahertz, fluorescence, computer vision, electronic nose, multispectral, ultrasound, X-ray) for the generic term “nondestructive”, because ScienceDirect caps the number of Boolean connectors per query.<br>**PubMed:**<br>(meat[tiab] AND (near-infrared OR hyperspectral OR Raman OR fluorescence OR "electronic nose" OR spectroscopy) AND (spoilage OR microbial OR "TVB-N" OR freshness OR adulteration) AND ("machine learning" OR "deep learning" OR chemometrics)), Filters: 2015-2026; English.
Selection process	8	Specify the methods used to decide whether a study met the inclusion criteria of the review, including how many reviewers screened each record and each report retrieved, whether they worked independently, and if applicable, details of automation tools used in the process.	Literature Search Strategy (p.3). A single reviewer screened titles/abstracts and full texts against the eligibility criteria with no automation tools used. These were later reviewed, verified, and supplemented by the co-authors and adjudicated by the corresponding author.
Data collection process	9	Specify the methods used to collect data from reports, including how many reviewers collected data from each report, whether they worked independently, any processes for obtaining or confirming data from study investigators, and if applicable, details of automation tools used in the process.	Literature Search Strategy (p. 3). A single reviewer extracted the data from the selected studies, capturing, for each study, the sensing modality, meat species and matrix, target attribute, acquisition mode, analytical/AI model, validation approach, reported performance metrics, and bibliographic reference. Study authors were not contacted, and no automation was used.
Data items	10a	List and define all outcomes for which data were sought. Specify whether all results that were compatible with each outcome domain in each study were sought (e.g., for all measures, time points, analyses), and if not, the methods used to decide which results to collect.	Outcome metrics (R^2, RMSE, RPD, accuracy) are defined in Table 2 (p. 12) and reported per study in Table 1 (pp. 11-12) and Table 3 (pp. 18-19).
	10b	List and define all other variables for which data were sought (e.g. participant and intervention characteristics, funding sources). Describe any assumptions made about any missing or unclear information.	Variables (meat species/matrix, sensing technique, target attribute, analytical/AI method, model performance, reference) are tabulated in Tables 1, 3 and 4.
Study risk of bias assessment	11	Specify the methods used to assess risk of bias in the included studies, including details of the tool(s) used, how many reviewers assessed each study and whether they worked independently, and if applicable, details of automation tools used in the process.	No formal risk-of-bias/quality-appraisal tool was applied.
Effect measures	12	Specify for each outcome the effect measure(s) (e.g. risk ratio, mean difference) used in the synthesis or presentation of results.	Reported. The effect/performance measures used are predictive-performance metrics (R^2, RMSE, RPD, accuracy), defined in Table 2 (p. 12) and reported per study in Tables 1 and 3. No pooled effect measure applies, as no meta-analysis was performed.
Synthesis methods	13a	Describe the processes used to decide which studies were eligible for each synthesis (e.g. tabulating the study intervention characteristics and comparing against the planned groups for each synthesis (item #5)).	Studies were grouped for synthesis by sensing technique (Section 3, pp. 4-24) and by AI method (Section 4, pp. 25-29).
	13b	Describe any methods required to prepare the data for presentation or synthesis, such as handling of missing summary statistics, or data conversions.	Not applicable: no numerical data conversion or handling of missing summary statistics (qualitative/narrative synthesis).
	13c	Describe any methods used to tabulate or visually display results of individual studies and syntheses.	Results are tabulated in Table 1 (spectroscopy studies, pp. 11-12), Table 3 (imaging studies, pp. 18-19), and Table 4 (cross-technique summary, p. 23), and visualised in Figures 1-2 and technique schematics (e.g., Figure 8, CNN).

Section and Topic	Item #	Checklist item	Location where item is reported
	13d	Describe any methods used to synthesize results and provide a rationale for the choice(s). If meta-analysis was performed, describe the model(s), method(s) to identify the presence and extent of statistical heterogeneity, and software package(s) used.	Not applicable - narrative synthesis; no meta-analysis was performed, given heterogeneity in sensing platforms, reference methods, and validation designs.
	13e	Describe any methods used to explore possible causes of heterogeneity among study results (e.g. subgroup analysis, meta-regression).	Not applicable: no meta-analysis performed
	13f	Describe any sensitivity analyses conducted to assess robustness of the synthesized results.	Not applicable: no meta-analysis performed
Reporting bias assessment	14	Describe any methods used to assess risk of bias due to missing results in a synthesis (arising from reporting biases).	Not applicable: no meta-analysis performed
Certainty assessment	15	Describe any methods used to assess certainty (or confidence) in the body of evidence for an outcome.	Not applicable - formal certainty rating was not applied
**RESULTS**			
Study selection	16a	Describe the results of the search and selection process, from the number of records identified in the search to the number of studies included in the review, ideally using a flow diagram.	Literature search (p. 4). Figure 2 (PRISMA flow diagram and narrative counts in the Literature Search Strategy, p. 3: 514 records identified -> duplicates removed -> title/abstract and full-text screening -> 148studies included; 20 foundational references added by citation tracking (168 sources total).
	16b	Cite studies that might appear to meet the inclusion criteria, but which were excluded, and explain why they were excluded.	Exclusion categories and counts are shown in Figure 2, p. 4 (destructive-only 14; no quantitative performance 16; non-meat matrix 10; review/duplicate dataset 7).
Study characteristics	17	Cite each included study and present its characteristics.	Included-study characteristics are presented in Table 1 (spectroscopy: meat type, attribute, acquisition mode, analytical approach, performance, reference; pp. 11-12), Table 3 (imaging; pp. 18-19), and Table 4 (cross-technique summary; p. 23), with narrative detail in Sections 3-4 (pp. 4-29).
Risk of bias in studies	18	Present assessments of risk of bias for each included study.	Not applicable: no risk-of-bias appraisal undertaken.
Results of individual studies	19	For all outcomes, present, for each study: (a) summary statistics for each group (where appropriate) and (b) an effect estimate and its precision (e.g. confidence/credible interval), ideally using structured tables or plots.	Per-study performance estimates (R^2, RMSE, RPD, accuracy) are given in Table 1 (pp. 11-12) and Table 3 (pp. 18-19), with supporting discussion in Sections 3-4 (pp. 4-29).
Results of syntheses	20a	For each synthesis, briefly summarise the characteristics and risk of bias among contributing studies.	Narrative synthesis is organised by sensing technique (Section 3, pp. 4-24) and AI approach (Section 4, pp. 25-29), summarising the contributing studies within each group.
	20b	Present results of all statistical syntheses conducted. If meta-analysis was done, present for each the summary estimate and its precision (e.g. confidence/credible interval) and measures of statistical heterogeneity. If comparing groups, describe the direction of the effect.	Not applicable: no statistical synthesis/meta-analysis performed.
	20c	Present results of all investigations of possible causes of heterogeneity among study results.	Not applicable: no meta-analysis.
	20d	Present results of all sensitivity analyses conducted to assess the robustness of the synthesized results.	Not applicable: no meta-analysis.
Reporting biases	21	Present assessments of risk of bias due to missing results (arising from reporting biases) for each synthesis assessed.	Not applicable: no meta-analysis.

PRISMA 2020 Checklist

Section and Topic	Item #	Checklist item	Location where item is reported
Certainty of evidence	22	Present assessments of certainty (or confidence) in the body of evidence for each outcome assessed.	Not applicable: no meta-analysis.
**DISCUSSION**			
Discussion	23a	Provide a general interpretation of the results in the context of other evidence.	Section 5 Challenges and Limitations (pp. 30-31), Future Research Directions (pp. 31-32), and Conclusion (p. 32) interpret the findings against deployment readiness and existing evidence.
	23b	Discuss any limitations of the evidence included in the review.	Section 5 (pp. 30-31) discusses instrumental, computational, and deployment limitations of the technologies.
	23c	Discuss any limitations of the review processes used.	The review process was not prospectively registered, and no formal risk-of-bias or certainty-of-evidence appraisal was performed.
	23d	Discuss implications of the results for practice, policy, and future research.	Future Research Directions (pp. 31-32) and Conclusion (p. 32) give implications for practice, industrial deployment, and future research.
**OTHER INFORMATION**			
Registration and protocol	24a	Provide registration information for the review, including register name and registration number, or state that the review was not registered.	This review was not registered
	24b	Indicate where the review protocol can be accessed, or state that a protocol was not prepared.	A formal review protocol was not prepared
	24c	Describe and explain any amendments to information provided at registration or in the protocol.	The review was not registered, and there were no protocol amendments.
Support	25	Describe sources of financial or non-financial support for the review, and the role of the funders or sponsors in the review.	Funding statement, p. 33: research grant no. PJ017041, Rural Development Administration, Republic of Korea. The funder had no role in the design, conduct, analysis, interpretation, or reporting of the review.
Competing interests	26	Declare any competing interests of review authors.	Competing interests are declared: author Yun-Kil Kwon was employed by XCore System Co.; the remaining authors declare none. Conflicts of Interest statement, p. 33 (lines 1011–1014).
Availability of data, code, and other materials	27	Report which of the following are publicly available and where they can be found: template data collection forms; data extracted from included studies; data used for all analyses; analytic code; any other materials used in the review.	All data supporting this review are contained within the article. The full literature-search strategies and the list of included studies with their extracted characteristics can be provided as supplementary materials. No new primary data or analytic code was generated in this review.

From: Page MJ, McKenzie JE, Bossuyt PM, Boutron I, Hoffmann TC, Mulrow CD, et al. The PRISMA 2020 statement: an updated guideline for reporting systematic reviews. BMJ 2021;372:n71. doi: 10.1136/bmj.n71. This work is licensed under CC BY 4.0. To view a copy of this license, visit https://creativecommons.org/licenses/by/4.0/
